# A morphine reward generalization mouse model based on conditioned place preference and aversion

**DOI:** 10.1002/brb3.2970

**Published:** 2023-03-30

**Authors:** Xue‐Fei Hou, Ya‐Bo Zhao, Yue‐Xiong Yang, Jing Zhu, Li‐Su Zhu, Lin Xu, Qi‐Xin Zhou

**Affiliations:** ^1^ School of Life Sciences Yunnan University Kunming Yunnan China; ^2^ Laboratory of Learning and Memory, Key Laboratory of Animal Models and Human Disease Mechanisms Kunming Institute of Zoology Kunming Yunnan China; ^3^ Yunnan Key Laboratory of Stem Cell and Regenerative Medicine, Biomedical Engineering Research Center Kunming Medical University Kunming Yunnan China

**Keywords:** conditional place preference, generalization, morphine, substance use disorders

## Abstract

**Background:**

Conditioned place preference (CPP) is a common behavioral paradigm for studying the association of unconditioned stimulus reward memory with context. Generalization is a flexible memory recall pattern developed on the basis of original memory. Drug‐seeking behaviors in substance use disorders (SUDs) exhibit diversity, which we generally attribute to the highly generalized features of SUD memory. However, to date, there are no animal models for SUD generalization studies.

**Methods:**

We design the generalization box (G‐box) and the generalization retrieval process based on the conditioned place preference (CPP) model. In the memory retrieval stage, we replaced the conditioning CPP box (T‐box) with a generalization box (G‐box) to study drug generalization memory. For appearance, the generalized boxes have different angles and numbers of sides compared to the conditioning boxes. For the visual cues, the shapes of the symbols are different (triangle icons for the hexagonal chamber and dot icons for the round chamber), but the orientation information remains the same. To establish CPP generalization, the mice received morphine on the vertical or horizontal side of a conditioning box (T‐box) and saline on the other side. Then, after CPP conditioning, the generalization test was performed in a generalization box (G‐box: hexagonal chamber and Gr‐box: round chamber) 21 days later.

**Results:**

CPP‐conditioned mice still displayed a clear preference for similar visual information in the G‐box. CPA‐conditioned mice behaved similarly to CPP, with mice consistently avoiding similar visual information in the G‐box. We further observed that the generalization results are similar using two generalization boxes (G‐box and Gr‐box).

**Conclusion:**

In this study, we succeeded in creating a simple and effective generalization model for morphine reward. The establishment of this model provides a new tool for generalization studies of SUD and therapy in humans.

## Graphical abstract

We use a behavioral paradigm based on conditional place preference to explore the generalization of drug memory. It provides a new approach for substance use disorders research.

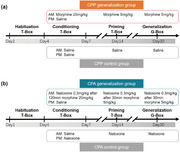

## 1 INTRODUCTION

Generalization is a phenomenon in which the same response is made to different but similar stimuli (Zhou et al., 2017). This phenomenon was first discovered by Pavlov. Pavlov observed that noise similar to a bell also caused salivation responses in dogs (Pavlov, 1927). Since it is difficult to return to a context that is completely consistent with memory, most memory retrieval in life belongs to memory generalization. Memories retrieved in different environments with similar cues are considered to be generalized, and similar environments have features such as shape (Zhou et al., 2017), sound (de Hoz & Nelken, 2014), and floor. Substance use disorders (SUDs) display strong drug craving and seeking behavior in similar cues in humans, often showing abnormal flexibility in drug‐seeking behavior, which is pathologically overgeneralized (Gandolphe & Nandrino, 2011; Kaczkurkin et al., 2017). In the early stage of drug use, a strong associative memory is formed between the drug and the context (Torregrossa & Taylor, 2016), and then the drug‐associated memories can be evoked by similar environments. Drug‐related memory is an obvious cause of SUD, which is a significant challenge in SUD treatment. Currently, generalization models based on conditioned fear memory are widely used in the study of posttraumatic stress disorder (Bergstrom, 2020; Rau et al., 2005). However, there is no generalized behavioral paradigm for substance abuse research.

Preclinical models of SUD are critical for elucidating the neurobiological mechanisms that contribute to drug‐related behaviors. Conditioned place preference (CPP) is a common behavioral paradigm for studying the association of unconditioned stimulus reward memory with context. It has been applied to rodent, primate, and human species (Linhardt et al., 2022; Lu et al., 2005; Wu et al., 2016) and is one of the common methods for studying SUDs. This model incorporates Pavlovian learning, memory, and motivated behavior (McKendrick & Graziane, 2020). CPP devices can also be used to study conditioned place aversion (CPA). CPA is usually used in studying drug withdrawal (Gracy et al., 2001). In the CPA experiment, animals avoided drug‐related contexts due to drug withdrawal and had lower CPP scores. CPP/CPA experiments typically consist of three phases: habituation, training (conditioning), and retrieval (postconditioning) (Cunningham et al., 2006; McKendrick & Graziane, 2020; Prus et al., 2009). In the habituation phase, the animals were mainly exposed to the CPP device to familiarize them with the context. During the conditioning phase, animals were trained alternately with the vehicle (on one side of the box) and the drugs (on the other side of the box) to associate the drug with the context. Animals associate the context with the drug, which is the basis for later drug‐seeking behavior performance (Mantsch et al., 2016; Wang et al., 2000). After a few days, animals were placed in the device to retrieve the drug memory, and the CPP score was used as an evaluation. Generally, animals in CPP experiments remain in the drug‐related context for a longer period of time, as evidenced by an increase in CPP scores. In contrast, animals in CPA experiments spent less time in the drug‐related context, as shown by a decrease in CPP scores. Drug priming, an effective method to retrieve the reinstatement of drug memory, has been used to model drug seeking and relapse (Aguilar et al., 2009; Deroche‐Gamonet, 2020; Kuhn et al., 2019; McKendrick & Graziane, 2020), a defining characteristic of SUD.

In our study, combined with the high generalization properties of human SUD, we improved the CPP process and proposed establishing a CPP generalization model. The CPP generalization model uses CPP equipment in the original laboratory, which is easy to obtain and provides a relevant model for the study of human generalization and SUD treatment.

## 2 METHODS

### 2.1 Drugs

Morphine is purchased from Northeast Pharmaceutical Group Shenyang No. 1 Pharmaceutical Co., Ltd. Naloxone hydrochloride dihydrate is purchased from Aladdin (N140482).

### 2.2 Animals

FVB/NJ mice, 12 weeks old, weighing approximately 18–22 g, were donated by Dr. Yu‐Qiang Ding and housed at the Kunming Institute of Zoology, Chinese Academy of Sciences. Animals were housed in groups of 4 in each cage. The ambient temperature was controlled at 22 ± 1°C, and the rearing followed a 12 h/12 h light/dark cycle with free access to food and water. The experimental procedures were approved by the Animal Ethics Committee of Kunming Institute of Zoology, CAS (SMKX‐SQ‐20200803‐132).

### 2.3 Setup CPP and generalization boxes (G‐box)

**FIGURE 1:**
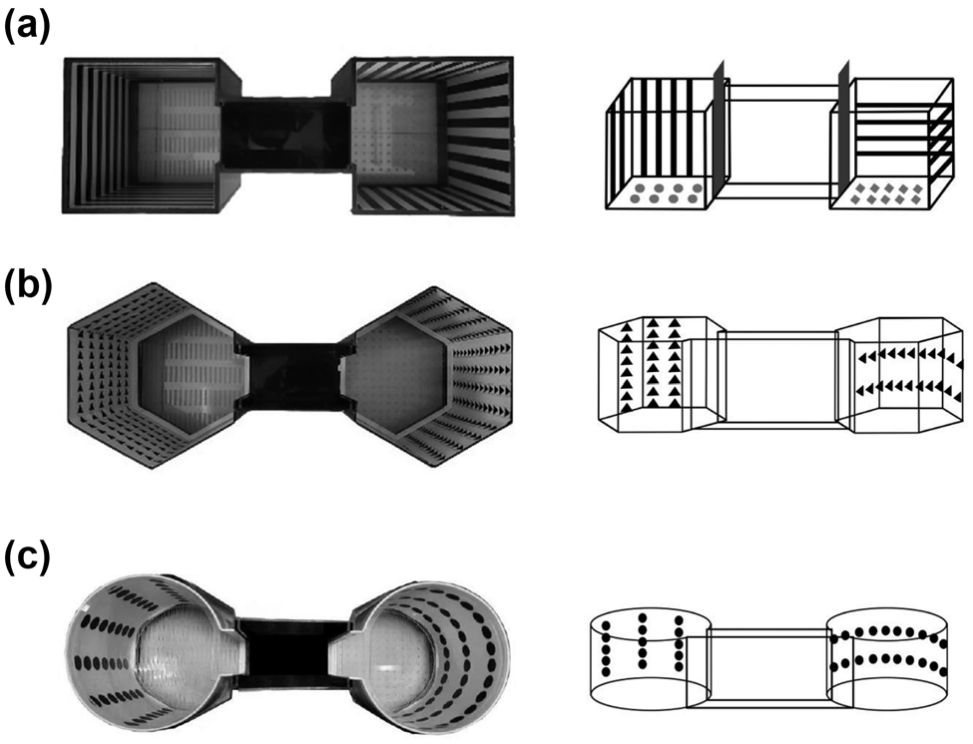
**The CPP box**. (a) Square box (T‐box), the box is square (dimensions: 20×20×20 cm), the channel is 20 cm long, each of two chambers has a movable door, and the chamber walls have different horizontal and vertical stripes (approximately 2 cm wide). (b) Hexagonal box (generalized box, G‐box). The box is similar in size to the square box, the channel length is 20 cm long, the two chambers have movable doors, and the wall has different horizontal and vertical icons (triangle, approximately 2 cm length). (c) Round box (generalized box, Gr‐box), the box is round (diameter: 20 cm), the channel length 20 cm long, each chamber with a movable door, walls with different horizontal and vertical icons ((circle, approximately 2 cm in diameter).

The CPP box is made of resin with three chambers. At both ends of the box are a square chamber (Figure 1a) (dimension: 20×20×20 cm), hexagon chamber (Figure 1b) (dimension: side length 13 cm, height 20 cm) or circle chamber (Figure 1c) (dimension: diameter 20 cm). The floor of the chambers contains many round or square holes (0.5 × 0.5 cm) (Figure 1d), and the pattern of the hole is different at each end of the chamber. We set the square chamber as the conditioning box (T‐box) and the hexagonal chamber and circular chamber as the generalization box (G‐box). There are two differences between the hexagonal (round box) and square boxes. First, the shape of the chamber is different, and the hexagonal (round) and square chambers are obviously different in angles and edges. Second, the visual cues in the chamber are different: triangle icons for the hexagonal chamber and dot icons for the round chamber. The icons are arranged horizontally and vertically. Visual cues are different in icons but similar in arrangement direction. Although all three different boxes contained floor cues, only the square conditioning box exposed the floor cues. The floor cues in round and hexagonal chambers are covered with white acrylic sheets or paper during the retrieval of generalized memory.

### 2.4 Statistical analysis

To assess contextual learning and memory in mice, we used the time mice spent on the morphine and morphine‐similar context minus time on the contralateral saline side as CPP scores for conditioned place preference, and a similar calculation method was used to calculate the CPP score of conditioned place aversion. GraphPad was used for data analysis, and a *t* test was used to compare the differences between the two groups; one‐way ANOVA and two‐way ANOVA were used for comparisons between multiple data sets. When *p* < .05, there was a significant difference, and the data are expressed as the mean ± SEM.

### 2.5 Process

#### 2.5.1 Handling

Mice were accustomed to the experimenter's touch and grip prior to the experiment. Generally, mice are placed in the laboratory 30 min beforehand each day, and the experimenter touches and grasps the mice a week in advance.

#### 2.5.2 Equipment setup

##### 2.5.2.1 Experimental environment

The laboratory room is quiet, odorless, and has weak light (e.g., reflected light from lamp lights on the wall). The light intensity of the box on both sides is measured by an illuminance meter, and the position of the box is adjusted so that the light intensity on both sides is consistent. Strong light and direct light should be avoided, but the light intensity should ensure the operation requirements of the experimental operators and video recording requirements.

##### 2.5.2.2 Data acquisition system

This experiment adopts a Noldus video tracking system, which contains computer and camera equipment. The camera equipment was installed approximately 2 m above the CPP box to obtain the movement speed and time spent in the box of the mice, and the data were exported into an Excel file.

#### 2.5.3 Conditional place preference (CPP) and conditional place aversion (CPA) generalization

##### 2.5.3.1 Habituation (Days 1–3) in square box (T‐box)

- Keep the channel closed and place the mice into the channel for 2 min.
- The channel was opened, and the mice were allowed to freely explore the box and the channel for 15 min.
- Mice were returned to their home cages.
- The feces were cleaned with paper towels, and the boxes or olfactory cues were cleaned with 75% alcohol.
- Repeated A–D on the second and third days, once a day.

##### 2.5.3.2 Conditioning (Days 4–7) in square box(T‐box)

Twenty‐four hours after habituation, experiments were completed between 9:00 am and 4:00 pm.

### 2.6 CPP conditioning

- The channel was kept closed, and the mice were placed in one of the conditioning chambers for 30 min immediately after morphine administration (20 mg/kg).
- The mice were returned to their home cages for 6 h.
- The feces were cleaned with paper towels, and the boxes or olfactory cues were cleaned with 75% alcohol.
- The channel was kept closed, and the mice were placed on the opposite side of the conditioning chamber for 30 min after administration of saline (the same dose as morphine).
- Mice were returned to their home cages.
- Repeated A–E for 3 days.

### 2.7 CPA conditioning

- The channel was kept closed, and the mice were placed in one side of the conditioning chamber for 30 min after saline administration.
- The feces were cleaned with paper towels, and the boxes or olfactory cues were cleaned with 75% alcohol.
- The mice were returned to their home cages for 4 h.
- Mice were given morphine (20 mg/kg) in their home cage 120 min before the next steps.
- The channel was closed, and the mice were placed in another side of the chamber for 30 min immediately after naloxone (0.3 mg/kg) administration.
- Mice were returned to their home cages.
- Repeated A–F for 3 days.

#### 2.7.1 After conditioning (memory retrieval day 14; generalization retrieval day 30)

- Priming in a square box (T‐box)

After the conditioning phase, the mice were returned to their cages for 7 days. Then, the priming procedure was implemented.

For CPP priming, the mice received morphine (5 mg/kg); for CPA priming, the mice received naloxone (0.3 mg/kg) 30 min after morphine (5 mg/kg) administration. The channel was kept open, and immediately after drug injection, the mice were placed into the box and allowed to freely explore the conditioning chambers and channel for 15 min.

- Generalization in hexagon (G‐box) or circle box (Gr‐box)

After the priming phase, the mice were returned to their cages and reared for at least 21 days. Then, the generalization procedure was conducted.

In CPP generalization, morphine was administered (5 mg/kg). In CPA generalization, naloxone was given (0.3 mg/kg) after 30 min of morphine (5 mg/kg). The channel was kept open, and the floor was flat in the generalization chamber. The mice were allowed to explore freely in the generalization box and the channel for 15 min immediately after drug injection.

#### 2.7.2 Control of CPP and CPA

##### 2.7.2.1 Habituation (Days 1–3) in square box(T‐box)

The process of the habituation phase is the same as that of the CPP habituation phase.

##### 2.7.2.2 Conditioning (Days 4–7) in square box(T‐box)

Twenty‐four hours after habituation, the experiment was completed between 9:00 am and 4:00 pm.

(A‐C) The three steps from A to C are the same as A–C in the CPP conditioning phase.
(D) The channel was kept closed and placed on the opposite side of the conditioning chamber for 30 min after giving the mice saline (or naloxone for CPA).
(E) The mice were returned to their home cages.
(F) Repeated A–E for 3 days.

##### 2.7.2.3 After conditioning (memory retrieval day 14; generalization retrieval day 30)

(A) Priming in a square box (T‐box)

After the conditioning phase, the mice were returned to their cages for 7 days. Then, the priming procedure was implemented.

For CPP priming, the mice received saline; for CPA priming, the mice received naloxone (0.3 mg/kg). The channel was kept open, and the mice were free to explore the conditioning chambers and channel for 15 min.

(B) Generalization in hexagon box (G‐box)

After the priming phase, the mice were returned to the cage and reared for at least 21 days. Then, the generalization procedure was conducted.

The mice were given saline (or naloxone for CPA). The channel was kept open, and the floor was flat in the generalization chamber. The mice were allowed to explore freely in the generalization chamber and the channel for 15 min immediately after drug injection.

### 2.8 Calculation of CPP score

In the CPP experiment, the time the mice spent in the morphine‐matched or morphine‐similar chamber minus the time spent in the contralateral chamber was used as the CPP score. In the CPA experiment, the time the mice spent in the naloxone‐matched or naloxone‐similar chamber minus the time spent in the contralateral chamber was used as the CPP score. Time in the channel for mice was not included in the calculation.

## 3 RESULT

### 3.1 Formation of generalization in CPP

**FIGURE 2:**
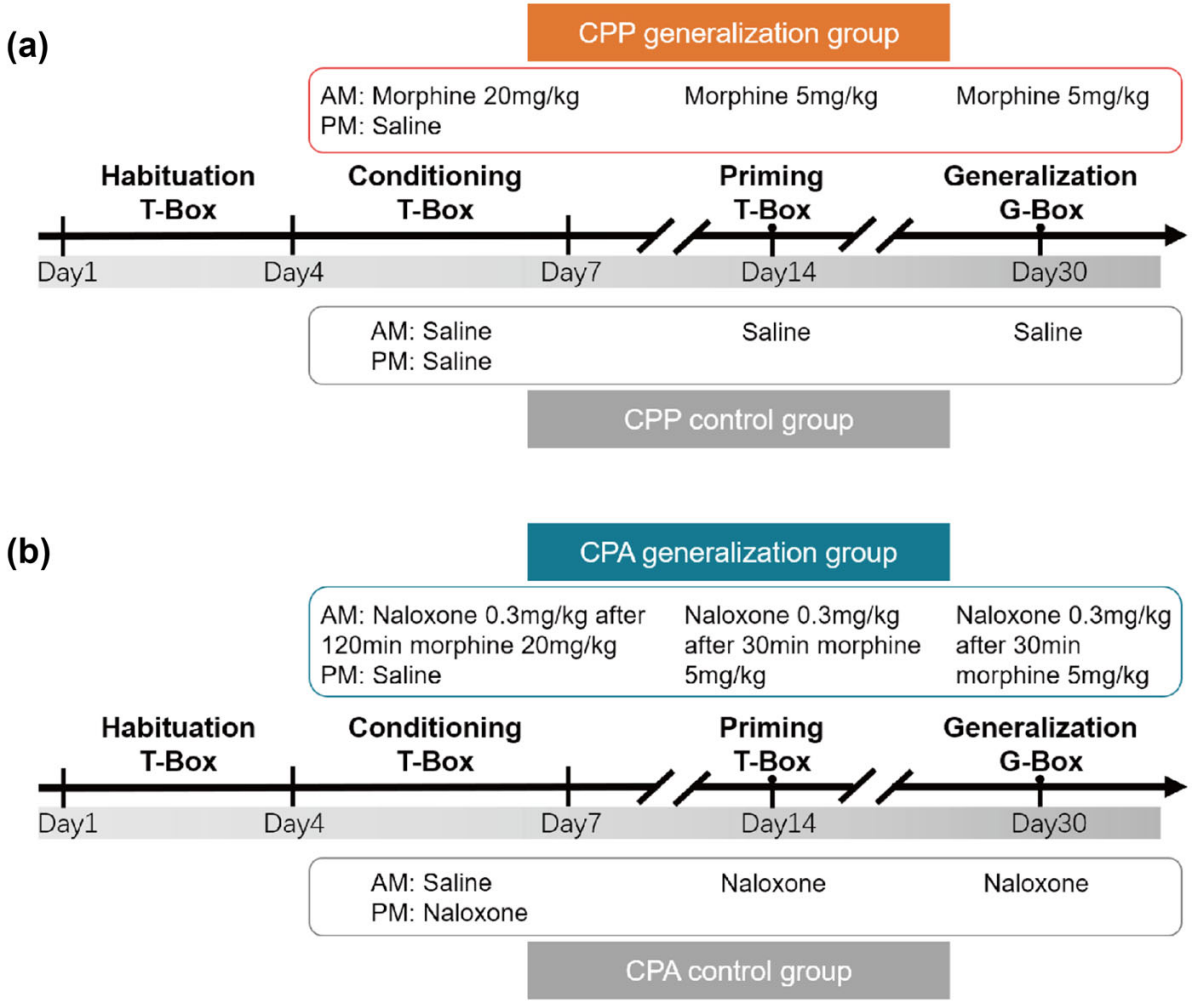
**Experimental design of the protocol for CPP and CPA**. (a) CPP experimental procedure. Kept the channel door open. Mice were habituated to 15 min per day for 3 days. After habituation, the channel door was closed for drug conditioning, mice were injected with 20 mg/kg morphine in the morning and placed in one side of the chamber for 30 min, and mice were injected with normal saline in the afternoon and placed on the other side of the chamber for 30 min. After drug conditioning, we waited 7 days for priming extraction and 21 days for generalization extraction. Keeping the channel open, the mice were injected with 5 mg/kg morphine, immediately placed in a box, and allowed to explore for 15 min. The chamber contained no floor cues for generalization. The procedure for the control group was similar to that of the CPP experiment, except that saline was used instead of morphine. (b) CPA experimental procedure. The procedure of the CPA experiment is similar to that of the CPP experiment. For conditioning, morphine (20 mg/kg) was administered 120 min before naloxone (0.3 mg/kg). At the priming and generalization phase, morphine (5 mg/kg) was administered 30 min before naloxone (0.3 mg/kg). The procedure for the control group was similar to that of the CPA experiment.

By placing mice into the square conditioning box (T‐box, horizontal and vertical stripes) and the hexagonal generalization box (G‐box, triangular icon arranged in a horizontal and vertical orientation), we measured conditioned memory (T‐box) and generalized memory (G‐box). The CPP generalization group (*n* = 9) was subjected to the CPP generalization procedure, and the CPP control group (*n* = 9) was subjected to the CPP control procedure (Figure 2a). The procedure in the CPP control group was the same as that in the CPP generalization group except that morphine was changed to saline.

**FIGURE 3:**
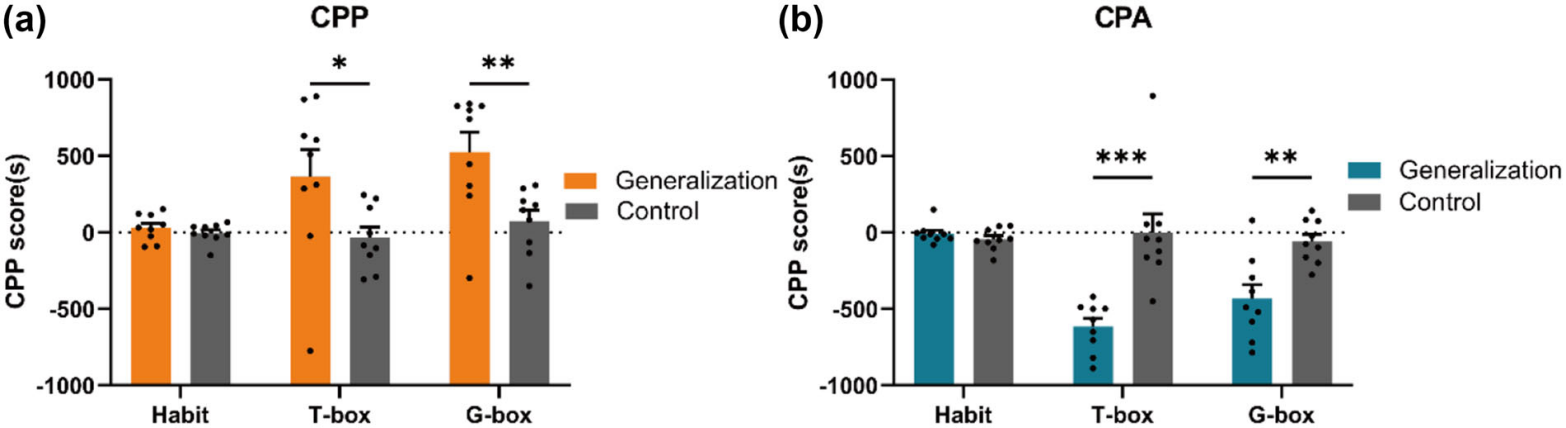
**Typical results of CPP and CPA priming and generalization**. (a) The CPP scores of the generalization group and the control group in the CPP experiment of three stages: habituation (Habit), priming (T‐box), and generalization (G‐box). *n* = 9, two‐way ANOVA. (b) The CPP scores of the generalization group and the control group in the CPA experiment at the three stages: habituation (Habit), priming (T‐box), and generalization (G‐box). *n* = 9, two‐way ANOVA. *p < .05, **p < .01, ***p < .001.

There was no difference between the CPP generalization group and CPP control group during the habituation phase, indicating that the mice did not show a clear preference for the chamber prior to conditioning. On day 14 of the priming test, mice in the CPP generalization group had higher CPP scores in the conditioning box (T‐box) than the CPP control group, suggesting that a stable morphine preference memory was established between morphine and the T‐box. On day 30 of the generalization test, mice in the CPP generalization group had significantly higher CPP scores in the generalization box (G‐box) (Figure 3a, *n* = 9, *F*(2,48) = 2.671, Habit *p* = .991, T‐box *p = .017, G‐box **p = .006, two‐way ANOVA) (Supplementary material S1). The results showed that mice could also extract high CPP scores from triangle stripes after forming high CPP scores with the context of strip patterns. The results indicate that the mice were able to recall and extract the stripe information in different contexts based on the drug‐conditioned memory, even though the stripe information itself was changed.

### 3.2 Formation of generalization in CPA

We further explored the generalization of mice in the CPA model. Animals avoided drug‐related contexts due to drug withdrawal and showed lower CPP scores in the CPA. The equipment and procedures adopted in CPA were similar to those in CPP. The CPA generalization group (*n* = 9) received the CPA generalization procedure, and the CPA control group (*n* = 9) received the CPA control procedure (Figure 2b). The procedure in the CPA control group was the same as that in the CPA generalization group except that morphine was changed to saline.

There was no difference between the two groups in the habituation period; on day 14 for priming, the CPP scores of the CPA generalization group were significantly lower in the conditioning chamber (T‐box) than the CPA control group, suggesting that the CPA generalization group developed aversive memory for the conditioning chamber (T‐box); on day 30 for generalization, CPP scores in the CPA generalization group were significantly lower in the generalization chamber (G‐box) than the CPA control group (Figure 3b, *n* = 9, *F*(2,48) = 11.40, Habit *p* = .976, T‐box ***p < .01, G‐box **p = .001, two‐way ANOVA) (Supplementary, S1). The results showed that the generalization group mice induced aversion memory in the generalization chamber (G‐box) of the triangle stripe. The results indicate that the mice were able to recall and retrieve the stripe information even when the stripe information changed. Therefore, we further confirmed that CPA generalization could be formed, which is similar to the result of CPP generalization. Combining the generalization results of CPP and CPA, we concluded that the CPP model can form a generalization.

### 3.3 Generalization exists universally in CPP

**FIGURE 4:**
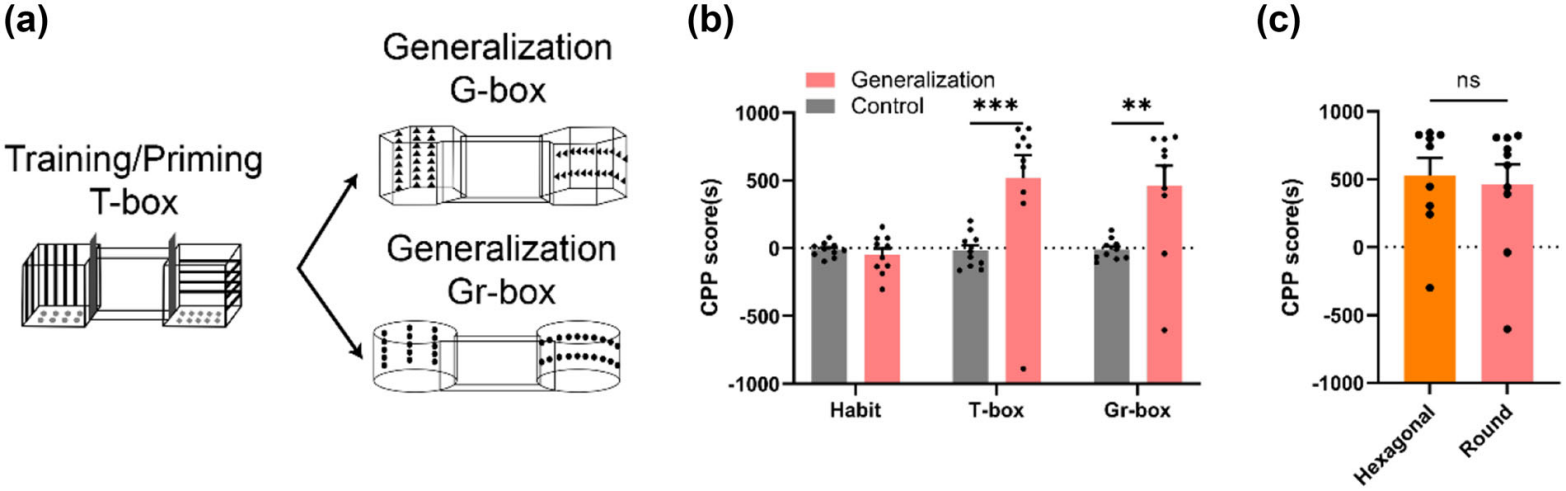
**Generalized in different contexts**. (a) Two different generalization boxes (G‐box and Gr‐box) are used for generalization after conditioning and priming in the square box (T‐box). (b) CPP scores for three stages: habituation (Habit), priming (T‐box), and generalization (Gr‐box). *n* = 10, two‐way ANOVA. (c) Comparison of CPP scores for generalization in hexagonal box (G‐box) and round box (Gr‐box), *n* = 10, *t* test. **p < .01, ***p < .001.

We observed that generalization can be formed in hexagonal boxes and continue to study the box and cue dependence of generalization memory. Therefore, we designed a round generalization box (Gr‐box) whose shape was quite different from the box shape used before and designed a dot icon arranged horizontally and vertically inside the round box (Figure 1c). For the generalization study in the round box, the CPP generalization group (*n* = 10) and CPP control group (*n* = 10) received similar habitation and priming phases in the square box (T‐box) and then underwent a generalization test in the round box (Gr‐box) (Figure 4a). The results were the same as those described before in the habitation and priming phases in the square box. On day 30, the CPP scores in the round box (Gr‐box) in the CPP generalization group were significantly higher than those in the CPP control group, indicating that mice could generalize in the round box (Figure 4b, *n* = 10, *F*(1,54) = 17.85, T‐box ***p ≤ .001, **p = .002, Gr‐box, two‐way ANOVA). We then compared the CPP scores of the round box (Gr‐box) and hexagonal box (G‐box) during the generalization phase, and there were no differences between the two boxes (Figure 4c, *n* = 10, *p* = .754, *t* test), showing that both boxes could form generalization. The results showed that although the cues in these boxes were different, generalized memory could be retrieved using cues similar to those in the initial morphine conditioning context, such as stripe orientation information.

In conclusion, the mouse generalization model has been successfully established, and the retrieval of this memory varies in the icon of the stripe and is similar in the cue direction. Based on our experimental results, it is speculated that the retrieval of generalized memory may rely mainly on similar cues. Generalization of drug memory exists universally even if the context changes significantly and demonstrates that our mouse generalization model is stable.

## 4 DISCUSSION

We successfully established a mouse model of morphine generalization by using the modified CPP model and generalization box. We designed a generalization box (G‐box), which differed from the condition box in shape and visual cues. We found that conditioned mice had higher CPP scores for the generalization box (G‐box) in morphine conditioning (CPP) and lower CPP scores on the generalization box (G‐box) in morphine‐naloxone conditioning (CPA). The results indicated that morphine generalization could be formed using the CPP procedure and CPP device. Furthermore, we compared the generalization ability between the Gr‐box and G‐box and found no significant difference in CPP generalization between the two boxes. The results showed that drug generalized memory retrieval does not depend on the shape features of the box but more on visual cues. In conclusion, we found that similar visual cues are one of the conditions that trigger drug memory generalization, providing support for further analysis of human drug memory.

Animal drug models are commonly used in preclinical research (Zanda & Fattore, 2018). SUD‐related research models are typically self‐administration models that examine decision‐making related to drug use and CPP models that examine contextual memory related to drug use (Green & Bardo, 2020; Groman et al., 2022; McKendrick & Graziane, 2020). Drug‐related memories are aberrantly reinforced in SUD populations, and their behavior toward drug seeking is variable and flexible, which largely depends on the generalizing effect of drug memory. Previous studies of conditioned place preference were based more on the precise retrieval of memories, and mice were able to establish stable memories between drugs and the context of use. SUD memory is evaluated by the degree of preference of the animal for the condition context in the CPP model (Suzuki, 1999). That is, although direct exposure and drug induction occurred in the original conditioning chamber, the mice were able to retrieve the associative memory between the drug and the context (Marszalek‐Grabska et al., 2021; McKendrick & Graziane, 2020; Napier et al., 2013; Scherma et al., 2021), and our experiments are consistent with these results. However, studies on drug generalization in mice have not been reported, and studies have shown that fear generalization in mice depends on the similarity of cued information, such as shape (Yu et al., 2021; Zhou et al., 2017), sound (Pollack et al., 2018) and floor (Yu et al., 2021). This experiment used visual information similar to cues for generalized memory retrieval, which is consistent with generalizations of fear memory. However, the drug memory in this experiment relies on similar visual cue direction information, which is different from the visual information used in the literature, and the mechanism by which cue information is used for drug generalization needs to be investigated in further experiments.

During the conditioning phase, mice established associative memories between drug use and the environment. Memory accumulates with the number of conditioning sessions. Drug memories were generally stable at the end of conditioning. However, withdrawal after continuous morphine use can trigger withdrawal symptoms (Fishbain et al., 1993). To exclude the effect of withdrawal symptoms on drug priming and considering the administration route and timing in this experiment, we performed drug priming on the 7th day after conditioning. Our results showed that mice showed a clear preference for the context on day 7 after conditioning in CPP/CPA, suggesting that cues in boxes can evoke drug‐related memory, which is an important method to evaluate the presence of drug memory (McKendrick & Graziane, 2020; Sanchis‐Segura & Spanagel, 2006). Several studies have shown that the formation of generalized memory takes 14 days or even longer (Hyman, 2005; Napier et al., 2013; Zhou et al., 2017). In addition, drug memory is a strong long‐term memory. Therefore, we performed a generalization test at least 21 days after conditioning and placed mice in a novel environment similar to the conditioning box to detect drug generalization. We found a clear preference in the CPP/CPA experiment, indicating drug memory recovery under similar cue activation (McKendrick et al., 2020; O'Brien et al., 1992). Therefore, our model can be an important tool for exploring the generalized memory of human SUD. In addition, previous studies have shown that generalization is time‐dependent and formed gradually (Pollack et al., 2018), and the time window and characteristics of cues in forming generalization need further study.

The generalization model is an effective animal model to simulate the generalization of human drug memory, which has a certain role in promoting the study of human drug memory. It is impossible for SUDs to live in exactly the same context every day, and we often see that the pursuit of drug use belongs to generalized memory retrieval (Zhou et al., 2017). Therefore, it is necessary to establish animal generalization models of human SUDs, which contribute to the study of drug generalized memory. Future experiments will allow us to further explore the cellular and molecular basis of the occurrence of drug generalized memory, which will be proposed in further studies.

In summary, we developed an animal model of drug generalization based on the modified CPP and CPA paradigms. This modified paradigm has been successfully applied to demonstrate drug generalization in mice. We also found that drug generalization relies more on directional information in cues than on the box itself. This paradigm is simple, easy to operate, requires no special devices, and can provide a model for advancing SUD generalization research.

## AUTHOR CONUTIBUTUONS

X.‐F.H. and Y.‐X.Y. designed the experiments. X.‐F.H., J.Z.,and L.‐S.Z. performed the behavioral experiments. Y.‐B.Z. wrote the paper. Q.‐X.Z. and L.X. supervised the project.

### PEER REVIEW

The peer review history for this article is available at https://publons.com/publon/10.1002/brb3.2970.

## Supporting information


**Figure S1. The CPP scores of CPP and CPA procedures in different groups**. (A) The CPP scores of the four groups (control (Gene)/naïve/control/generalization) in the CPP experiment of three stages: habituation (Habit), priming (T‐box), and generalization (G‐box). *n* = 9, two‐way ANOVA. (b) The CPP scores of the four groups (control (Gene)/naïve/control/generalization) in the CPA experiment at the three stages: habituation (Habit), priming (T‐box), and generalization (G‐box). *n* = 9, two‐way ANOVA. *p < .05, **p < .01, ***p < .001.
