# Supplementary material for: A morphine reward generalization mouse model based on conditioned place preference and aversion
Source: Brain Behav. 2023 Mar 30;13(5):e2970. doi: 10.1002/brb3.2970 (PMC10175973; doi:10.1002/brb3.2970)
Supplement: Supplementary file 1 — Figure S1. The CPP scores of CPP and CPA procedures in different groups. (A) The CPP scores of the four groups (control (Gene)/naïve/control/generalization) in the CPP experiment of three stages: habituation (Habit), priming (T‐box), and generalization (G‐box). n = 9, two‐way ANOVA. (b) The CPP scores of the four groups (control (Gene)/naïve/control/generalization) in the CPA experiment at the three stages: habituation (Habit), priming (T‐box), and generalization (G‐box). n = 9, two‐way ANOVA. *p < .05, **p < .01, ***p < .001. [file BRB3-13-e2970-s001.docx]

Supplementary

S1: **
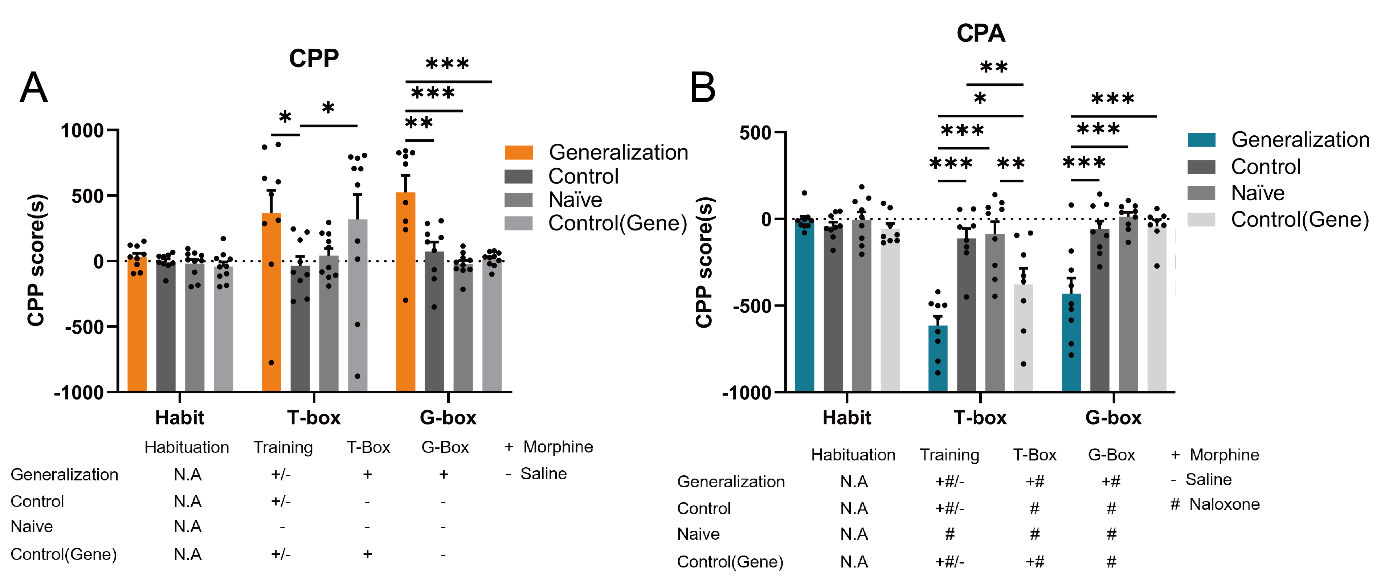
**

**Figure S1. The CPP scores of CPP and CPA procedures in different groups.**

(A) The CPP scores of the four groups (control (Gene)/naïve/control/generalization) in the CPP experiment of three stages: habituation (Habit), priming (T-box), and generalization (G-box). n=9, two-way ANOVA. (B) The CPP scores of the four groups (control (Gene)/naïve/control/generalization) in the CPA experiment at the three stages: habituation (Habit), priming (T-box), and generalization (G-box). n=9, two-way ANOVA. *P < 0.05, **P < 0.01, ***P < 0.001.
